# Left ventricular non-compaction as a potential source for cryptogenic ischemic stroke in the young: A case-control study

**DOI:** 10.1371/journal.pone.0237228

**Published:** 2020-08-14

**Authors:** Pauli Pöyhönen, Jouni Kuusisto, Vesa Järvinen, Jani Pirinen, Heli Räty, Lauri Lehmonen, Riitta Paakkanen, Nicolas Martinez-Majander, Jukka Putaala, Juha Sinisalo

**Affiliations:** 1 Heart and Lung Center, Helsinki University Hospital and Helsinki University, Helsinki, Finland; 2 Department of Clinical Physiology and Nuclear Medicine, HUS Medical Imaging Center, Helsinki, Finland; 3 Radiology, HUS Medical Imaging Center, University of Helsinki and Helsinki University Hospital, Helsinki, Finland; 4 Neurology, Helsinki University Hospital and University of Helsinki, Helsinki, Finland; Universitatsklinikum Wurzburg, GERMANY

## Abstract

**Background:**

Up to 50% of ischemic strokes in the young after thorough diagnostic work-up remain cryptogenic or associated with low-risk sources of cardioembolism such as patent foramen ovale (PFO). We studied with cardiac magnetic resonance (CMR) imaging, whether left ventricular (LV) non-compaction—a possible source for embolic stroke due to sluggish blood flow in deep intertrabecular recesses—is associated with cryptogenic strokes in the young.

**Methods:**

Searching for Explanations for Cryptogenic Stroke in the Young: Revealing the Etiology, Triggers, and Outcome (SECRETO; NCT01934725) is an international prospective multicenter case-control study of young adults (aged 18–49 years) presenting with an imaging-positive first-ever ischemic stroke of undetermined etiology. In this pilot substudy, 30 cases and 30 age- and sex-matched stroke-free controls were examined with CMR. Transcranial Doppler (TCD) bubble test was performed to evaluate the presence and magnitude of right-to-left shunt (RLS).

**Results:**

There were no significant differences in LV volumes, masses or systolic function between cases and controls; none of the participants had non-compaction cardiomyopathy. Semi-automated assessment of LV non-compaction was highly reproducible. Non-compacted LV mass (median 14.0 [interquartile range 12.6–16.0] g/m^2^ vs. 12.7 [10.4–16.6] g/m^2^, p = 0.045), the ratio of non-compacted to compacted LV mass (mean 25.6 ± 4.2% vs. 22.8 ± 6.0%, p = 0.015) and the percentage of non-compacted LV volume (mean 17.6 ± 2.9% vs. 15.7 ± 3.8%, p = 0.004) were higher in cases compared to controls. In a multivariate conditional logistic regression model including non-compacted LV volume, RLS and body mass index, the percentage of non-compacted LV volume (odds ratio [OR] 1.55, 95% confidence interval [CI] 1.10–2.18, p = 0.011) and the presence of RLS (OR 11.94, 95% CI 1.14–124.94, p = 0.038) were independently associated with cryptogenic ischemic stroke.

**Conclusions:**

LV non-compaction is associated with a heightened risk of cryptogenic ischemic stroke in young adults, independent of concomitant RLS and in the absence of cardiomyopathy.

**Clinical trial registration:**

SECRETO; NCT01934725. Registered 4^th^ September 2013. https://clinicaltrials.gov/ct2/show/NCT01934725

## Introduction

There is growing evidence that the global incidence of ischemic stroke in young adults (18–50 years) has substantially increased since the 1980s [1]. Along with individual tragedy, early-onset stroke accounts for a considerable socioeconomic impact due to long life expectancy of these patients. Therefore, effective treatment and prevention strategies of strokes in the young are of uttermost importance. However, up to 50% of ischemic strokes in young adults are cryptogenic, i.e. without evident causality or associated with low-risk cardioembolic sources after standard diagnostic evaluation [2–4]. leading to untargeted secondary prevention. Furthermore, in these circumstances, the patient and proxies are left with uncertainty regarding the nature and prognosis of such an event.

It has been suggested that most cryptogenic ischemic strokes are thromboembolic by nature, having sources such as proximal arteries or heart, or venous sources via right-to-left shunts [5]. The concept of embolic stroke of undetermined source (ESUS) was the first attempt to systematically define this patient population, characterized by a non-lacunar brain infarct without proximal occlusive arterial stenoses or major-risk cardioembolic source [6]. Patent foramen ovale (PFO) is the most frequent low-risk cardiac finding in young patients with cryptogenic ischemic stroke [2]. Based on most recent evidence, paradoxical embolism is the likely cause for the stroke in many patients with PFO. However, a good proportion of these PFOs may still represent a bystander without causal relation to the stroke and further research on the mechanisms of PFO-related and PFO-nonrelated strokes are warranted [7]. Our present study includes both ESUS (+) and ESUS (-) cryptogenic stroke patients.

We hypothesized that there would be subtle structural or functional cardiac abnormalities detectable by cardiac magnetic resonance (CMR) imaging as possible novel sources for cryptogenic strokes in the young beyond standard cardiac imaging work-up. Left ventricular (LV) non-compaction (LVNC) has been suggested as a rare cause of embolic stroke due to sluggish blood flow in deep intertrabecular recesses [8, 9]. CMR imaging has shown good reproducibility to estimate the global non-compacted LV mass [10–12]. In this pilot case-control study, we studied whether non-compacted LV mass, the ratio of non-compacted to compacted LV mass, and the percentage of non-compacted LV volume evaluated with CMR are associated with cryptogenic ischemic stroke in the young.

## Methods

### Study population

Searching for Explanations for Cryptogenic Stroke in the Young: Revealing the Etiology, Triggers, and Outcome (SECRETO; NCT01934725) is an international prospective multicenter case-control study of young adults (age 18–49) presenting with an imaging-positive first-ever ischemic stroke of undetermined etiology. The main study protocol has been published in more detail previously [13]. Briefly, patients are included after standardized and timely diagnostic procedures, including brain magnetic resonance imaging, imaging of intracranial and extra-cranial vessels with either computed tomography angiography or magnetic resonance angiography, and cardiac imaging to rule out established causes of ischemic stroke. The routine cardiac work-up included standardized transthoracic (TTE) and transesophageal echocardiography (TEE) with bubble test [14], transcranial Doppler (TCD) ultrasound with bubble test, 12-lead ECG, and at least 24-hour Holter ECG. Those with PFO-related strokes were included in the study. Patients were age- and sex-matched to stroke-free controls in a 1:1 fashion. Controls were searched randomly through population registers. A list of 20 potential controls per one patient were identified and invited with a letter one by one. In few cases, when the aforementioned method was not successful, patients’ nonrelated proxies or proxies of the study personnel served as controls. This study complies with the Declaration of Helsinki. Written informed consent was obtained from all study participants. SECRETO has been approved by the Ethics Committee of Helsinki and Uusimaa Hospital District.

In this single center substudy conducted in Helsinki University Hospital, 30 cases and 30 controls were examined with CMR and TCD bubble test with inclusion period between December 2013 and May 2017. Consecutive patients enrolled in the main study, and control subjects matched (for age ± 5 years and sex) to these patients, were asked to participate in the study. Detailed clinical history was recorded from all study subjects, including actual blood pressure, height and weight. Patients were classified as ESUS (+) and ESUS (-). Admission National Institute of Health Stroke Scale (NIHSS) score were also recorded in patients as a measure of stroke symptom severity. None of the patients had PFO closure before CMR.

### CMR protocol

All subjects were imaged using a 1.5T Avanto^fit^ magnetic resonance imaging system (Siemens Healthcare, Erlangen, Germany). A 32-channel body receiver coil was used in combination with ECG-gating. Gated three-direction localizer was used as a basis for image acquisition. Specific technical parameters for each sequence type used are reported in Supporting information (S1 Table).

Half-Fourier-acquisition single-shot turbo spin echo (HASTE) sequence was first acquired in transaxial planes covering the entire heart. The LV long axis was determined in this transaxial set of sections using the built-in three-point plane planning tool. The LV two-chamber (2CH) view was defined with points in LV apex, and both caudal and cranial center of mitral ring. Balanced steady-state free precession (bSSFP) cine images were then acquired, with cartesian sampling in 2CH view, followed by short-axis (SAX) cine images covering the entire heart. Three-chamber (3CH), four-chamber (4CH) and right-ventricular outflow tract (RVOT) views were acquired last before contrast agent administration.

Gadolinium-based contrast agent (gadoterate meglumine, Dotarem) of 0.2 mmol/kg was used as a contrast agent. After contrast agent administration, through plane phase contrast (PC) flow images were acquired for ascending aorta and pulmonary artery. Inversion time (TI) scout was then acquired to probe the most suitable TI for phase-sensitive inversion recovery (PSIR). Ten to fifteen minutes after contrast administration late gadolinium enhancement (LGE) images were acquired for the entire LV in SAX direction, and in 2CH, 3CH and 4CH directions.

### CMR analysis

All images were analyzed by CMR-trained physicians (J.K., V.J., H.R., P.P.) blinded to case-control status and all clinical information. LV volumes and mass were evaluated using standard protocols [15]. The presence of LGE was visually assessed and required the visibility in two orthogonal planes. Image analysis was performed using QMass MR software® (version 8.1, Medis Medical Imaging Systems, Leiden, the Netherlands). Results are given as absolute values and as indices standardized to body surface area (BSA) using the Mosteller formula [16].

**Fig 1:**
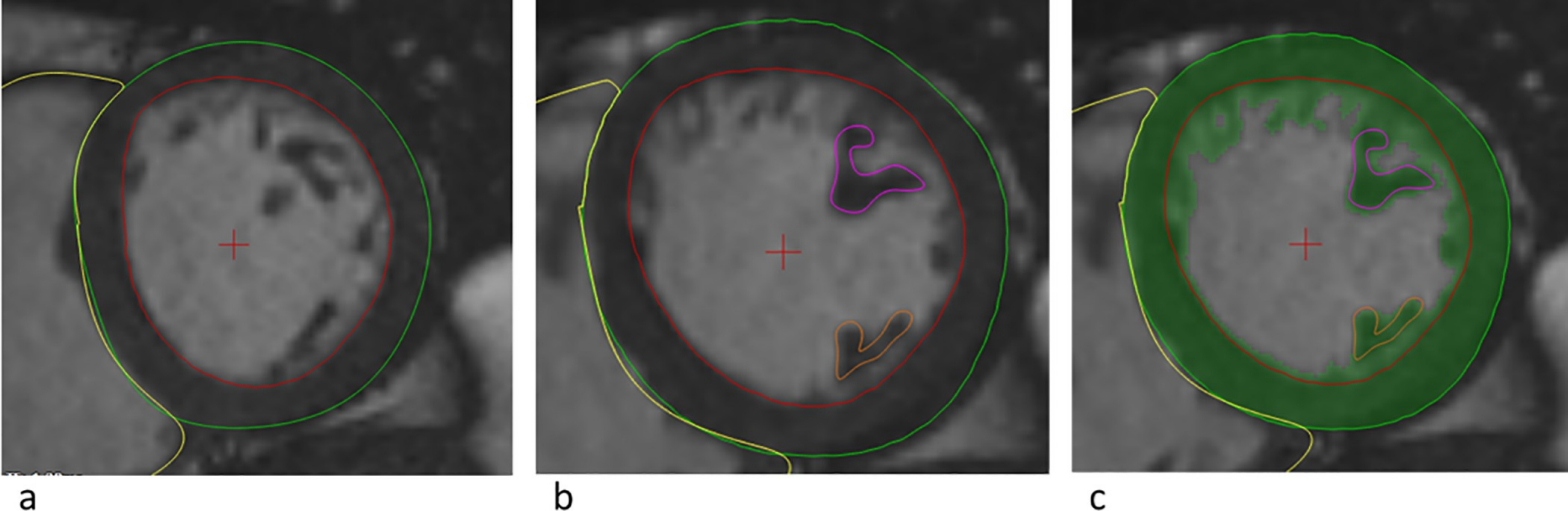
Quantification of left ventricular (LV) non-compacted mass. a) QMass software was used to define LV epicardial (green line) and endocardial (red line) borders. These borders were manually adjusted after automatic quantification. b) Papillae were manually quantified (pink and orange lines). c) MassK mode activated: the algorithm applies to areas inside the epicardial borders, and defines myocardium (green color) according to pixel intensities automatically according to the set threshold. Non-compacted LV mass was achieved by subtracting compacted LV mass from the total LV mass (green color). Compacted LV mass included both LV wall (areas between green and red lines) and papillary muscles (areas within pink and red lines). These analyses were made for all short axis cine end-diastolic slices that contained LV except the most basal slices.

Non-compacted and compacted LV masses were measured by a single observer (J.K.) using a similar semi-automatic method as previously presented [10, 11]. Measurements were independently repeated in twenty random subjects by another researcher (P.P.) to assess inter-observer reproducibility. The whole semi-automatic quantification process of LVNC is presented in Fig 1. MassK mode of Qmass software was used to automatically segment blood and muscle tissue based on pixel intensities, with the nominal threshold of the algorithm. Basal slice or slices containing both LV and mitral valve were excluded to avoid incorrect detection of mitral valve structures as trabeculated myocardium. Thus, we accepted the resulted underestimation of LV mass and volume for this analysis. Papillary muscles were included in compacted LV mass and excluded from end-diastolic LV volume. In addition to non-compacted LV mass, we determined the ratio of non-compacted to compacted LV mass and the percentage of non-compacted LV volume of end-diastolic LV volume.

### Transcranial Doppler bubble test

Status of right-to-left shunt (RLS) was evaluated with TCD bubble test in both cases and controls. TCD was performed according to consensus guidelines in unilateral insonation of middle cerebral artery in semisupine position [17]. Agitated 10 mL saline-blood solution was injected into the right antecubital vein. The test was performed at rest and with at least once with well-performed Valsalva maneuver. The presence of RLS (≥3 microbubbles) and highest degree of RLS were recorded. Moderate-to-severe RLS (≥10 microbubbles), was considered clinically significant, consistent with recent consensus statement [7].

### Statistical analysis

Normality of continuous variables was tested using the Shapiro-Wilk test. Continuous variables are presented as mean ± standard deviation for normally distributed variables or median (interquartile range) for non-normally distributed variables, and categorical variables as frequency (%).

In case-control analyses, comparison between paired dichotomous variables was performed with McNemar’s test, between normally distributed continuous variables with repeated measures Student’s t-test, and between non-normally distributed continuous variables with Wilcoxon signed rank test. Conditional logistic regression allowed assessing the association of non-compacted LV volume with stroke adjusting for the presence of RLS and body mass index (predefined model). Since moderate-to-severe RLS was not eligible for conditional logistic regression, comparison between patients with and without moderate-to-severe RLS was performed with independent samples t-test.

To study inter-observer reproducibility, Bland-Altman analysis [18] was performed and intraclass correlation coefficients calculated by using a two-way mixed model for consistency. A *p*-value of <0.05 was considered statistically significant and all tests were 2-sided. Statistical analysis was performed on SPSS 25 statistical package (SPSS, Chicago, IL).

## Results

### Clinical characteristics

The median age of cases at stroke onset was 41 (35–44) years and of stroke-free controls at study inclusion 42 (35–45) years (p = 0.131). Half of both cases and controls were women. Patients were taller, they had higher body weight, and larger BSA. There were no significant differences in cardiovascular risk factors between the groups (Table 1). Neither cases nor controls had previous known cardiovascular diseases, such as coronary artery disease, atrial fibrillation, heart failure, or peripheral artery disease. At least moderate RLS was more frequent in cases compared to controls (40% vs. 3%, p = 0.001). The median NIHSS score of cases was 1.5 (0–4) and 70% of cases fulfilled the ESUS criteria.

**Table 1:** Baseline characteristics of young adults with cryptogenic stroke and stroke-free controls.

	Cases (n = 30)	Controls (n = 30)	*P*-value
**Demographics and body measurements**			
Age at stroke onset or at inclusion, years	41 (35–44)	42 (35–45)	0.131
Gender, female	15 (50)	15 (50)	1.000
Height, cm	175 ± 9	171 ± 9	0.013
Weight, kg	87 ± 18	79 ± 17	0.042
Body surface area, m2	2.04 ± 0.24	1.93 ± 0.24	0.022
Body mass index, kg/m2	28.4 ± 5.0	26.9 ± 5.2	0.294
**Cardiovascular risk factors**			
Hypertension*	9 (30)	4 (13)	0.227
Diabetes type 1	1 (3.3)	0 (0)	1.000
Any diabetes	1 (3.3)	0 (0)	1.000
Current smoking	9 (30)	11 (36.7)	0.774
Alcohol week doses†	2 (1–4)	4 (2–6)	0.416
Physical inactivity	4 (13)	4 (13)	1.000
**Right-to-left shunt††**	19 (63.3)	12 (40.0)	0.065
**Right-to-left shunt, at least moderate††**	12 (40.0)	1 (3.3)	0.001
**Stroke characteristics**			
Fulfills ESUS criteria	21 (70.0)	NA	
NIH Stroke Scale	1.5 (0–4)	NA	

Values are mean ± standard deviation, median (interquartile range) or number (%)

*Hypertension on any criteria (office hypertension > 150/90, history of hypertension, or blood pressure medication prior stroke)

†One control had missing data.

††Evaluated with transcranial Doppler (n = 59) and transesophageal echocardiography (n = 1)

ESUS, Embolic Stroke of Undetermined Source; NA, not applicable; NIH Stroke Scale—National Institute of Health Stroke Scale.

### Basic structures and function

The mean duration from stroke to CMR was 31 ± 12 months (median 28, interquartile range 22–41, range 14–52). There were no significant differences in LV volumes, masses or stroke volumes between cases and controls (Table 2). Also, aortic and pulmonary artery stroke volumes obtained by PC flow measurements were similar between the groups. Four of 30 cases had atrial septal aneurysm compared to none of the controls (p = 0.125). Pulmonary vein status was similar between groups. All study subjects had visually normal LV shape, morphology and trabeculation, and only one case had visible LGE (p = 1.000).

**Table 2:** Cardiac magnetic resonance of young adults with cryptogenic stroke and stroke-free controls: Basic structures and function.

	Cases (n = 30)	Controls (n = 30)	*P*-value
LV end-diastolic volume index, ml/m^2^	82.5 (77.9–89.3)	84.5 (76.0–93.5)	0.910
LV end-systolic volume index, ml/m^2^	30.5 ± 6.0	31.1 ± 7.2	0.734
LV stroke volume index, ml/m^2^	54.0 ± 7.0	55.0 ± 10.3	0.666
LVEF, %	64.1 ± 4.7	63.8 ± 5.1	0.820
LA volume max index, ml/m^2^	44.5 ± 6.7	44.7 ± 8.3	0.907
Aortic stroke volume*, ml	99.0 ± 16.2	97.5 ± 17.9	0.730
Pulmonary artery stroke volume*, ml	93.7 (83.4–106.3)	93.5 (87.6–106.2)	0.863
Atrial septal aneurysm	4 (13.3)	0 (0.0)	0.125
Pulmonary vein status abnormality†	6 (20.0)	5 (16.7)	1.000

Values are mean ± standard deviation, median (interquartile range) or number (%)

*Only 29 cases had flow measurements (results of 29 case-control pairs)

† Normal pulmonary veins defined as four separate returning veins to left atrium.

LA, left atrial; LV, left ventricular; LVEF, left ventricular ejection fraction.

### Left ventricular non-compaction

Non-compacted and compacted LV masses were measured with a high degree of inter-observer reproducibility (Table 3).

**Table 3:** Inter-observer reproducibility of non-compaction measurements in 20 subjects.

	Observer 1 Mean (SD)	Observer 2 Mean (SD)	Blandt-Altman Mean (SD)	ICC (95% CI)	*P*-value
Non-compacted LV mass, g/m^2^	14.1 ± 3.9	14.4 ± 4.3	-0.3 ± 1.5	0.937 (0.847–0.974)	<0.001
Compacted LV mass, g/m^2^	59.0 ± 14.2	58.7 ± 13.0	0.3 ± 2.8	0.988 (0.970–0.995)	<0.001
Non-compacted to compacted LV mass ratio, %	24.1 ± 5.0	24.6 ± 5.1	-0.5 ± 2.9	0.833 (0.626–0.930)	<0.001
Non-compacted LV volume, % of EDV	16.4 ± 2.5	16.6 ± 3.1	-0.3 ± 1.4	0.883 (0.729–0.952)	<0.001

Values are mean ± standard deviation

CI, confidence interval; EDV, end-diastolic volume; LV, left ventricular end-diastolic volume; ICC, intraclass correlation coefficient; SD, standard deviation.

Non-compacted LV mass was greater in patients than in controls (median 14.0 [12.6–16.0] g/m^2^ vs. 12.7 [10.4–16.6] g/m^2^, p = 0.045) (Table 4). We also observed both a higher ratio of non-compacted to compacted LV mass (25.6 ± 4.2% vs. 22.8 ± 6.0%, p = 0.015) and a higher percentage of non-compacted LV volume (17.6 ± 2.9% vs. 15.7 ± 3.8%, p = 0.004) in cases compared to controls.

**Table 4:** Cardiac magnetic resonance of young adults with cryptogenic stroke and stroke-free controls: Left ventricular non-compaction analysis.

	Cases (n = 30)	Controls (n = 30)	*P*-value
Non-compacted LV mass, g	30.0 ± 7.4	26.4 ± 10.1	0.014
Non-compacted LV mass index, g/m^2^	14.0 (12.6–16.0)	12.7 (10.4–16.6)	0.045
Compacted LV mass, g	116.1 (95.7–135.8)	110.0 (93.4–127.7)	0.229
Compacted LV mass index, g/m^2^	55.3 (50.7–65.2)	57.1 (52.8–63.7)	0.797
Non-compacted to compacted LV mass ratio, %	25.6 ± 4.2	22.8 ± 6.0	0.015
Non-compacted LV volume, % of EDV	17.6 ± 2.9	15.7 ± 3.8	0.004

Values are mean ± standard deviation or median (interquartile range).

Papillary muscles are included in compacted LV mass.

EDV, end-diastolic volume; LV, left ventricular.

In a predefined multivariate conditional logistic regression model including non-compacted LV volume, the presence of RLS and body mass index, non-compacted LV volume (odds ratio [OR] 1.55, 95% confidence interval [CI] 1.10–2.18, p = 0.011) and RLS (OR 11.94, 95% CI 1.14–124.94, p = 0.038) were independently associated with cryptogenic ischemic stroke (Table 5).

**Table 5:** Conditional logistic regression: Risk factors for cryptogenic ischemic stroke in young adults.

	Univariate			Multivariate*		
	OR	95% CI	*P*-value	OR	95% CI	*P*-value
Right-to-left shunt, Yes/No	4.50	0.97–20.83	0.054	11.94	1.14–124.94	0.038
Non-compacted LV volume, % of EDV	1.39	1.07–1.79	0.013	1.55	1.10–2.18	0.011
Body mass index, kg/m2	1.06	0.95–1.17	0.299	0.965	0.845–1.103	0.602

Values are mean ± standard deviation

*Predefined model.

CI, confidence interval; EDV, end-diastolic volume; LV, left ventricular end-diastolic volume; OR, odds ratio.

There were no significant differences in LVNC parameters between patients with or without at least moderate RLS (Table 6).

**Table 6:** Left ventricular non-compaction in patients (n = 30) with and without moderate-to-severe right-to-left shunt (RLS).

	RLS- (n = 18)	RLS+(n = 12)	*P*-value
Non-compacted mass, g/m2	14.1 ± 2.2	15.3 ± 3.4	0.260
Compacted LV mass, g/m2	57.9 ± 9.6	57.2 ±9.3	0.846
Non-compacted to compacted mass ratio, %	24.8 ± 4.2	26.8 ± 4.0	0.197
Non-compacted volume, % of EDV	17.3 ± 3.4	18.0 ± 2.2	0.538

Values are mean ± standard deviation or median (interquartile range).

EDV, end-diastolic volume; LV, left ventricular.

## Discussion

**Fig 2:**
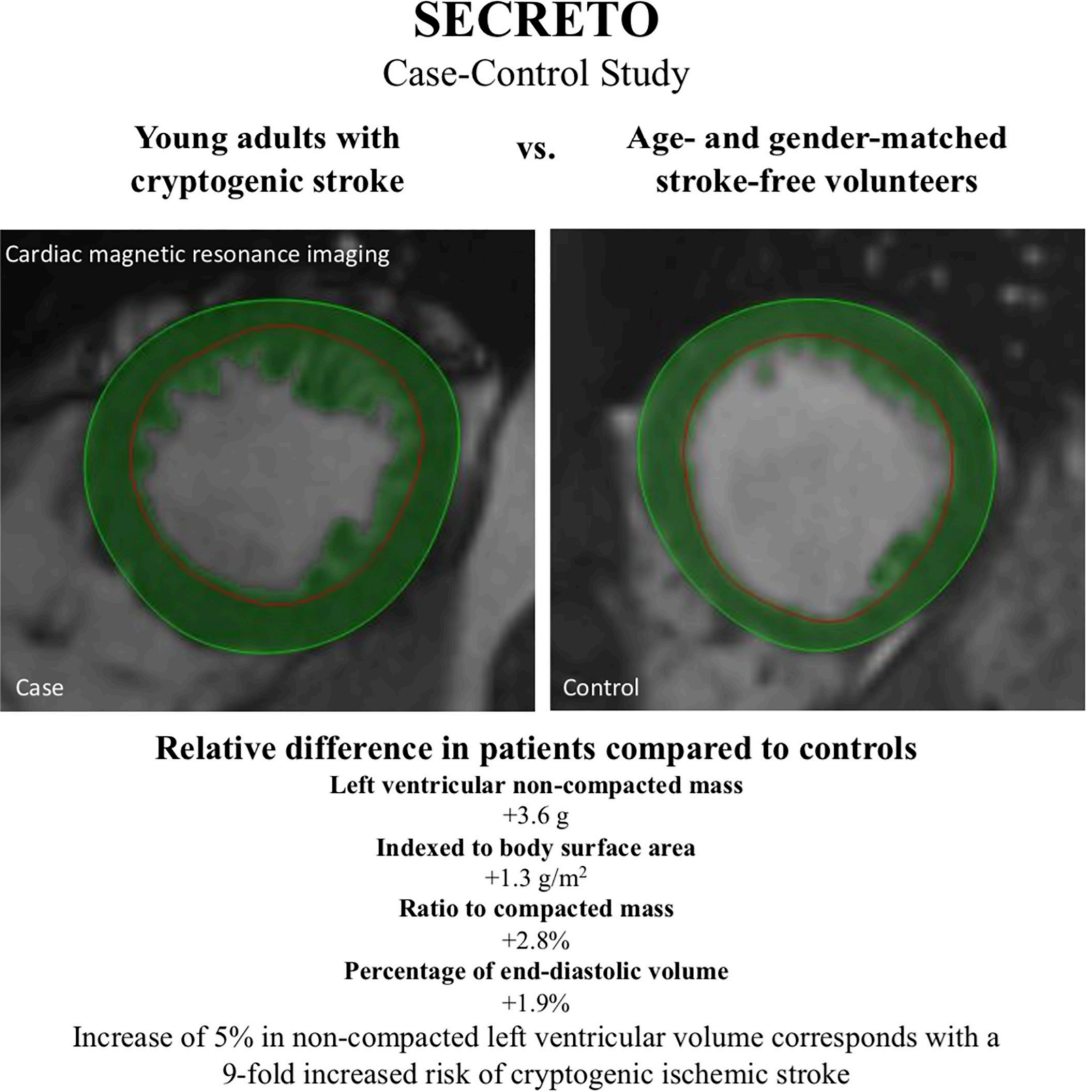
Increased left ventricular non-compaction may be associated with cryptogenic ischemic stroke in the young.

To the best of our knowledge, this is the first prospective case-control study to demonstrate an association between LVNC and cryptogenic ischemic stroke in young adults. The main finding of this study was that the percentage of non-compacted LV volume was higher in young adults with cryptogenic ischemic stroke compared to stroke-free controls of similar age and gender, independently of the presence of RLS. Moreover, non-compacted LV mass and the ratio of non-compacted to compacted LV mass were higher in patients compared to controls (Fig 2).

Heart failure, arrhythmias and systemic thromboembolic events are major clinical manifestations in LVNC [19–21]. As such, the LVNC can be caused by a genetic disorder of the myocardium, pathological loading conditions or physiological adaptation of the LV [22, 23]. The prevalence of LVNC has been highly variable, depending on the used imaging modality, study cohort and diagnostic criteria [24]. It has been controversial whether LVNC is an independent risk factor for stroke, or if embolism occurs mainly with atrial fibrillation or systolic dysfunction, both frequently associated with LVNC. In a retrospective case-control study of 62 patients with LVNC and 62 controls matched with age, gender and systolic function, LVNC itself was not associated with stroke or embolism [25]. In that study, LVNC was detected by echocardiography, which is less sensitive compared to CMR, and the selection process of the controls was unclear. In two other retrospective analyses, it was concluded that stroke is a rare complication of LVNC without other risk factors [8, 9]. In a recent meta-analysis, the risk of thromboembolism in patients with LVNC was similar to patients with dilated cardiomyopathy [26]. In our study, we observed an association between increased trabeculated myocardial tissue and cryptogenic ischemic stroke in young adults. The median non-compacted LV mass was 1.3 g/m^2^ higher, the mean non-compacted to compacted LV mass ratio 2.8% higher, and the percentage of trabeculated LV volume 1.9% higher in patients compared to stroke-free controls. Although these changes are relatively small, altogether 70% of our cases fulfilled the ESUS criteria and it has been presented that patients with ESUS have on average minor strokes consistent with small emboli [25].

Altogether 40% of our patients had at least moderate RLS, consistently with previous knowledge that PFO is a frequent low-risk cardiac finding in young adults with cryptogenic stroke [2]. However, a good proportion of PFOs may still represent a bystander [7]. In our study, 5% increase in non-compacted LV volume resulted in 9-fold risk (OR) of cryptogenic stroke, adjusted for the presence of RLS. Thus, the association between LVNC and cryptogenic stroke seemed to be independent of RLS, strengthening the hypothesis that there are also other RLS-nonrelated cardiac functional and structural factors increasing the likelihood for cryptogenic stroke in the young.

Assessment of the global trabecular LV mass has shown good reproducibility in previous studies [10–12]. Both our patients and controls had similar non-compacted LV masses compared to healthy subjects in an earlier study [27]. but higher compared to two other studies [10, 11]. The probable explanation for this difference is that our semi-automated method included more inter-trabecular space to non-compacted mass, as CMR does not differentiate between thin intertrabecular recesses from trabecular mass, and small inter-trabecular space is generally hard to quantify with cardiac imaging [21, 28]. Nevertheless, as we hypothesized, this inter-trabecular volume may serve as a source for thrombosis formation.

All of our study subjects had visually normal LV shape, morphology and trabeculation, and normal LV systolic function. In previous studies, isolated LVNC cardiomyopathy has been frequently associated with myocardial fibrosis, present in approximately half of the patients [29, 30], and fibrosis has been related to clinical disease severity and systolic dysfunction [29]. Only one of our patients had local fibrosis, detected by LGE, but otherwise normal LV morphology with normal volumes and function. Thus, our study subjects did not have LVNC cardiomyopathy phenotype. Earlier, it has been speculated whether increased trabeculation in healthy individuals is a pre-phenotype of underlying cardiovascular disease and adverse outcome. However, in a large population-based study of asymptomatic individuals followed by 9.5 years with repeated CMRs, there was no decline in LV function related to excess non-compaction [31].

According to a recent meta-analysis, CMR detects more readily LVNC changes compared to echocardiography, but the clinical significance of these changes is unclear [24]. Our finding suggests that, increased LVNC may be associated with cryptogenic stroke even if systolic function is normal.

As a major strength, our case-control study was based on prospectively enrolled patients with thorough baseline characterization for both cases and controls, including structural and standardized echocardiography protocol [13, 14]. Another strength is that the semi-automatic assessment of non-compacted LV mass was highly reproducible, probably due to only minor manual corrections. A limitation of this study is that the number of patients was modest. However, this was a pilot study with an extensive CMR imaging protocol, which has not been previously used in the patient population of interest, and we had no ruling hypothesis of the optimal sample size. This study gives information for future studies on optimal imaging sequences, use of imaging time, and optimal sample size. Although we matched controls for age and gender, and indexed parameters for BSA, we had limited possibilities to further adjust for confounders. Thus, the findings of this study should be verified in a larger patient cohort. Also, we were not able to define a cut-off value for non-compacted LV mass predisposing for cryptogenic stroke, as the assessment of global trabecular mass is method-dependent and we excluded the most basal LV slices for the consistency of measurements. Furthermore, we did not assess local non-compacted to compacted LV wall thickness ratio, frequently used to define isolated LVNC in CMR [32], but the global non-compacted LV mass which has shown high reproducibility in earlier studies [10–12].

## Conclusions

We displayed an association between increased LVNC and cryptogenic ischemic stroke in the young, independent of concomitant RLS and in the absence of cardiomyopathy, suggesting that trabeculation may be involved in thrombosis formation and embolic events in these patients.

## Supporting information

S1 Table Cardiac magnetic resonance protocol sequence parameters. (DOCX)

S1 File Secreto LVNC data. (SAV)

S2 File Secreto LVNC InterObserver 060420. (SAV)
