# Supplementary material for: Left ventricular non-compaction as a potential source for cryptogenic ischemic stroke in the young: A case-control study
Source: PLoS One. 2020 Aug 14;15(8):e0237228. doi: 10.1371/journal.pone.0237228 (PMC7428175; doi:10.1371/journal.pone.0237228)
Supplement: S1 Table — (DOCX) [file pone.0237228.s001.docx]

| **Table S1.** Cardiac magnetic resonance protocol sequence parameters | | | | | | | | | | | | | | | | | | |
| --- | --- | --- | --- | --- | --- | --- | --- | --- | --- | --- | --- | --- | --- | --- | --- | --- | --- | --- |
| **Sequence** | **Acquisition** | **ECG** | **RESP** | **Slice thickness (mm)** | **FOV read (mm)** | **FOV phase (%)** | **Base resolution** | **Phase resolution (%)** | **PE steps** | **Flip angle** | **PE direction** | **TR (ms)** | **TE (ms)** | **Averages** | **Slice spacing (mm)** | **ETL** | **Bandwidth (Hz/px)** | **Other** |
| 3-direction localizer | 2D | prosp | BH | 8 | 400 | 100 | 240 | 66 | 206 | 64 | ROW | 301,3 | 1,1 | 1 | 11,2 | 1 | 1155 |  |
| HASTE, TRA | 2D | prosp | BH | 8 | 370 | 75 | 256 | 75 | 85 | 160 | ROW | 612 | 39 | 1 | 8 | 56 | 780 |  |
| Cine, cartesian acquisition (2CH, 3CH 4CH, LV SAX, RVOT) | 2D | retro | BH | 6 | 320 | 81 | 192 | 80 | 220 | 55 | ROW | 33,7 | 1,2 | 1 | 0 | 1 | 930 | 40 cine phases |
| PC flow, through plane | 2D | retro | BH | 8 | 340 | 68,75 | 192 | 100 | 180 | 20 | COL | 48,4 | 2,63 | 1 | - | 1 | 450 | 40 cine phases, VENC 100-150 |
| TI scout, TRA | 2D | prosp | BH | 8 | 340 | 81,25 | 192 | 50 | 117 | 30 | ROW | 34,5 | 1,1 | 1 | - | 1 | 965 |  |
| PSIR (2CH, 3CH, 4CH, SAX) | 2D | prosp | BH | 8 | 300 | 76,25 | 160 | 100 | 194 | 40 | COL | 826,4 | 1,1 | 1 | 10 | 1 | 1200 |  |
| BH, breath hold; ECG, electrocardiogram; ETL, echo train length; FOV, field of view; HASTE, Half-Fourier-acquisition single-shot Turbo spin echo; LV, left ventricular; PC, phase contrast; PE, phase encoding; prosp, prospective; PSIR, phase-sensitive inversion recovery; RESP, respiration; retro, retrospective; RVOT, right ventricular outflow tract; SAX, short-axis; TE, echo time; TI, inversion time; TR, repetition time; TRA, transaxial; VENC, velocity encoding; 2CH, two chamber; 3CH, three chamber; 4CH, four chamber. | | | | | | | | | | | | | | | | | | |
